# Characterization of Persistent Virus-Like Particles in Two Acetate-Fed Methanogenic Reactors

**DOI:** 10.1371/journal.pone.0081040

**Published:** 2013-11-22

**Authors:** I-Chieh Chien, John Scott Meschke, Heidi L. Gough, John F. Ferguson

**Affiliations:** 1 Department of Civil and Environmental Engineering, University of Washington, Seattle, Washington, United States of America; 2 Department of Environmental and Occupational Health Sciences, University of Washington, Seattle, Washington, United States of America; University of Illinois, Urbana-Champaign, United States of America

## Abstract

The objective of this study was to characterize the morphology, size-distribution, concentration and genome size of virus-like particles (VLPs) in two acetate-fed *Methanosaeta*-dominated reactors to better understand the possible correlation between viruses and archaeal hosts. The study reactors were dominated by a single genus of acetoclastic methanogen, *Methanosaeta*, which was present at 6 to 13 times higher than the combined bacterial populations consisting of *Proteobacteria*, *Firmicutes*, and *Bacteroidetes*. Epifluorescent microscopy showed VLPs concentration of 7.1 ± 1.5×10^7^ VLPs/ml and 8.4 ± 4.3×10^7^ VLPs/ml in the two laboratory reactors. Observations of no detectable import of VLPs with the reactor feed combined long operational time since the last inocula were introduced suggests that the VLP populations were actively propagating in the reactors. Transmission electron microscopy images showed VLPs with morphology consistent with *Siphoviridae* in both reactors, and VLPs with morphologies consistent with *Myoviridae* in one of the reactors. The morphology, size-distribution and genome size of VLPs were distinct between reactors suggesting that unique viral populations inhabited each reactor, though the hosts of these VLPs remain unclear.

## Introduction

Acetoclastic methanogens inhabit a variety of natural ecosystems such as paddy field soils [1], fresh water and marine sediments [2,3] and acidic fens [4]. They are among the most important methane producers on earth, since two-thirds of the biogenic methane released to atmosphere is thought to be derived from the methyl group of acetate [5,6].

Most methanogens use hydrogen and carbon dioxide as substrates (hydrogenotrophic methanogens) while only two known genera of methanogens, *Methanosaeta* and *Methanosarcina*, are able to utilize acetate (acetoclastic methanogens). Because they use the same substrate, acetate, *Methanosarcina* and *Methanosaeta* compete with each other using different growth strategies. Having a higher growth rate (k) and lower acetate affinity (higher *K*s), *Methanosarcina* usually dominate in environments with high acetate concentrations. In contrast, with lower growth rate and higher acetate affinity, *Methanosaeta* is generally dominant when acetate concentrations decrease. *Methanosaeta* is ubiquitous and dominant in major biogenic methane releasing environments and have been postulated to be the predominant methane producer on earth [5].

Acetoclastic methanogens are also essential in engineered systems such as anaerobic digestion. Anaerobic digestion is used worldwide to treat organic waste generated from municipal, industrial and agricultural sources. Stability of anaerobic digestion has been investigated and improved over several decades [7–10]. Nevertheless, upsets and failures of the process still occur with no obvious explanation. Digestion failure is usually characterized by a decreased rate of methane production, accumulation of acetic and short chain length organic acids, and decreased pH [11], which implies the loss of functional microorganisms involved in the last step of anaerobic digestion, which is methanogenesis.

Viruses of Bacteria (bacteriophages or phages) and Archaea (archeoviruses) [12], have been shown to influence the composition of microbial communities through cell lysis [13]. By targeting the most rapid growing populations (“kill the winner” theory), viruses are believed to stimulate microbial diversity [14]. Diverse and abundant virus-like particles (VLPs) have been reported to inhabit anaerobic digestion systems, suggesting that VLPs may play an important role in functioning of this treatment process [15,16]. About two-thirds of the methane produced in mesophilic anaerobic digesters is from acetoclastic methanogens [17,18], and *Methanosaeta* is typically the dominant genus [19], which suggesting *Methanosaeta* may be a favorable target for viral attack. Therefore, viral attack on the *Methanosaeta* may explain observed process upsets characterized by loss of methane production. Viruses of acetoclastic methanogens have not been isolated, a task which is complicated by slow host growth rates (4.8 day doubling time for *Methanosaeta*) [20] and lack of anaerobic solid media methods for plaque assays (i.e. one month of *Methanosaeta* growth results in <1 mm diameter colonies and no lawn formation [21]).

The goals of this study were (1) to investigate the occurrence and persistence of viruses in two *Methanosaeta*-dominated reactors (2), to examine the morphology, size distribution and genome length of observed viruses, and (3) to determine the concentration of viruses in relation to acetoclastic methanogens and bacteria using two previously-established acetate-fed methanogenic enrichment reactors.

## Materials and Methods

### Reactors Operation and Analyses

The study reactors consisted of two semi-continuous stirred tank reactors (CSTR) that were originally established in 2002, as described by Conklin et al [22]. The reactors were inoculated with anaerobic digester sludge from the West Point Treatment Plant in Seattle, WA, fed aseptic Reduced Anaerobic Mineral Media (RAMM) supplemented with vitamins and acetate (234mM) as the sole carbon and energy source, and operated at 30-34°C. One reactor was fed in a single daily dose (daily-fed reactor) and the other in hourly increments (hourly-fed reactor). The daily-fed reactor was re-inoculated in 2007 and 2008 due to community population shift from *Methanosarcina* to *Methanosaeta*. But reestablishment of *Methanosarcina* population did not succeed. Table 1 summarizes the operating conditions for the reactors during the current study. During the study period, the reactors were fed 150 ml/day sterile feed and the same volume was manually wasted daily from each reactor. Due to a lower operating reactor volume in the daily-fed reactor during the study period, this reactor had a lower hydraulic retention time (HRT) (13.3 days) than the hourly-fed reactor (20 days).

**Table 1:** Experimental reactor characteristics.

Parameter	daily-fed reactor	hourly-fed reactor
reactor volume	2 L	3 L
vol. of media fed per day	150 ml	150 ml
hydraulic retention time	13.3 days	20 days
date of inoculation	March 2008	January 2002
pH	7.3	7.4
CH_4_ % in headspace	54%	56%

Gas production by the reactors was continually recorded by wet test meters (Precision Scientific, Chicago, IL). The concentrations of methane and carbon dioxide in headspace gas were analyzed by Carle Series 100A gas chromatograph equipped with a thermal conductivity detector (GC-TCD, Chandler Engineering, Tulsa, OK). The concentration of acetate in the reactor effluent was analyzed using a Shimadzu GC-2010 equipped with flame ionization detector (GC-FID, Shimadzu Scientific Instruments, Columbia, MD) and a DB-FFAP column (122-3232, Agilent Technologies, Santa Clara, CA).

### Autofluorescence Microscopy

The reactor effluents were examined by laser scanning confocal microscopy (Leica SP5 II, Leica Microsystems GmbH, Wetzlar, Germany) under the following conditions: excitation (405 nm laser) and emission (430-500 nm), which are used to visualize methanogens using their natural autofluorescent characteristics [23].

### Transmission Electron Microscopy (TEM)

VLPs in the reactors were viewed using transmission electron microscope (TEM, Philips CM100 TEM, 100kV). TEM samples were prepared in general accordance with the methods described by Ackermann [24]. Samples were filtered through 0.2 µm low protein binding polyethersulfone (PES) syringe filters (Whatman, Clifton, NJ). Filtrate was placed in a 10 ml-polycarbonate tube (Seton Scientific, CA) and ultracentrifuged at 171,500×g and 4°C for 2 h (Beckman Coulter 70.1 Ti rotor, Fullerton, CA). The viral pellet was resuspended in 1 ml of 0.1 M ammonium acetate and centrifuged again at 79,302×g and 4°C for 1 h. The pellet was resuspended into 0.2 ml of 0.1 M ammonium acetate and stored at 4°C.

To prepare TEM grids, 3 μl of poly-L-lysine was placed on a 300 mesh carbon-stabilized formvar-coated copper grids (TedPella Inc, Reading, CA) for 1 min. Then the poly-L-lysine was removed by wicking using Whatman no. 1 filter paper. Viral suspension (5 μl) was placed on the grid for 2 min. Finally, 1μl of negative stain, phosphotungstic acid (PTA, 2%, pH 7.2) was added for 1 min, and the grid was wicked dry. Prepared grids were placed in a desiccator at least overnight until examination by TEM. The head diameter and tail length of VLPs were measured by ImageJ (version 1.43u). Micrographs were screened, and clear images of VLPs were selected for measurement of VLP size.

### Epifluorescence Microscopy (EFM)

0.2 µm filtrate for EFM was collected daily for one week. Filtrate was harvested from both reactors: 0.5 h before and 4 h after the feeding of the daily-fed reactor (5 samples before and 5 samples after feeding from the daily-fed reactor); 3 samples before (0.5 h) and 4 samples after (0.5 h) the feeding from the hourly-fed reactor.

Nucleic acid stained VLPs were directly enumerated using Leica DM-LB microscope (Buffalo Grove, IL). VLPs were enumerated using EFM methods described by Noble and Fuhrman [25] with minor modification. Reactor samples were preserved in 10 mM sodium pyrophosphate, 2% formaldehyde and 94.5 mM of NaCl (final concentrations); preservation solutions used were prefiltered using a 0.02 µm Whatman Anotop™ syringe filter. Fixed solutions were held on ice for 15 min and filtered through Whatman 0.2 µm PES filters. Two µl of 1,000× SYBR Gold (Invitrogen Molecular Probes, Eugene, OR) was added to stain viral nucleic acid. After vortexing, samples were incubated on ice in the dark for at least 15 min. The stained VLPs were collected on 0.02 µm pore-sized Anodisc^TM^ aluminum oxide-membrane filters (6809-6002, Whatman, Clifton, NJ). After drying the filter on a Kimwipe^®^ tissue (Kimberly-Clark Corp., Roswell, GA) in the dark, the filter was placed on a glass slide and covered with a cover slip with freshly made mounting solution (50% glycerol and 50% PBS with 0.1% p- phenylenediamide). Slides were stored at -20°C until enumeration. For enumeration of VLPs, at least 10 images per filter were captured by digital camera (Leica DC 300F) using Image-Pro Plus 5.1 (Media Cybernetics, Inc., Bethesda, MD). Fields were randomly selected, avoiding the area near the supporting ring of the filter. Images were processed by IrfanView (version 4.27) (converted to grey images). VLPs were enumerated using ImageJ (version 1.43u).

### Pulsed Field Gel Electrophoresis (PFGE)

Cellular materials in 1 L of sample from each reactor were initially separated from viruses by centrifuging at 3696×g and 4°C for 30 min in a Sorvall Legend RT tabletop centrifuge equipped with a swinging-bucket rotor (Cat. No. 75006441) (Thermo Scientific, Waltham, MA). Supernatant was collected and serially filtered through 0.45 and 0.20 µm pore size low protein binding polyethersulfone (PES) syringe filters (6780-2502 and 6780-2504, Whatman, Clifton, NJ). Viruses in the filtrates were concentrated using a series of centrifugal ultrafiltration units (Centricon UFC703008, Amicon Ultra-15 UFC903024 and Amicon Ultra-0.5 UFC503008; Millipore, Billerica, MA) following the manufacturer’s instructions. In addition, ultrafilter retentates were washed twice in SM buffer (100 mM NaCl, 8 mM MgSO_4_·7H_2_O, 50 mM Tris-Cl; without gelatin) and the final volume adjusted to 50 µl. The concentrate was treated with 1U of DNase I (AM2222, Life Technologies, Grand Island, NY) at 37°C for 1 h to degrade free DNA followed by 65°C for 15 min and the addition of 10 mM of EDTA (final concentration) to inactivate the DNase I.

Equal volumes of concentrate and 2% low-melting agarose (A9414, Sigma-Aldrich, St. Louis, MO) were mixed by gentle pipetting and transferred to plug molds. After solidification, plugs were incubated overnight in extraction buffer (100 mM EDTA, pH 8.0, 1% SDS and 1 mg/ml proteinase K (4333793, Life Technologies, Grand Island, NY)) at room temperature with gentle mixing on a rocker. The plugs were rinsed three times with 1X TE buffer for 30 min and stored at 4°C. Plugs and the DNA marker (N3551S, New England Biolabs, Ipswich, MA) were placed into wells of a 1% agarose gel (162-0137, BioRad, Hercules, CA) prepared in 0.5X TBE (wells were sealed with 1% agarose). PFGE was performed with CHEF-DR II System (BioRad, Hercules, CA) using the following conditions: 0.5X TBE, 6 V/cm, 15°C for 24 hours, switch times ramped from 1-25 seconds. The gel was stained with SYBR Green I (Molecular Probes, Eugene, OR) for 30 min and the image was acquired by a UVP GDS-8000 System (UVP, Upland, CA).

### DNA Extraction

Genomic DNA from the reactors was isolated by UltraClean Soil DNA Isolation Kit (Mo Bio Laboratories, Carlsbad, CA). DNA extraction was performed following the manufacturers' protocol except the samples were disrupted using a FastPrep^®^-24 bead beater (MP Biomedicals, Solon, OH) at setting 5 m/s for 20 s.

### Quantitative PCR (qPCR)

Quantification of 16S rRNA genes of *Methanosaeta*, *Methanosarcina* and Bacteria were performed using an Eppendorf Mastercycler^®^ ep realplex and RealMasterMix kit (Eppendorf, Hauppauge, NY). Standards were prepared using PCR products of 16S rRNA genes of *Methanosaeta concilii* (DSM 6752), *Methanosarcina barkeri* (DSM 804) and *Sphingopyxis* TrD1 (GenBank^®^ accession number: JN940802), which were cloned using TOPO TA Cloning Kit (Invitrogen Molecular Probes, Grand Island, NY). Plasmids were isolated by QIAprep Spin Miniprep Kit (Qiagen, Valencia, CA) and quantified using a NanoDrop 1000 spectrophotometer (NanoDrop Technologies, Wilmington, DE). Plasmids were linearized by restriction enzyme EcoRI (R6011, Promega Co., Madison, WI). Reaction mixes contained 2 μl DNA templates, 4.5 μl RealMasterMix and individual forward and reverse primers adjusted with water to 10 μl. Primers used for qPCR [26,27] are summarized in Table 2. For qPCR of *Methanosaeta*, thermal cycling conditions were as follows: initial denaturation at 95°C for 10 min, 50 cycles with denaturation for 10 s at 95°C, annealing for 20 s at 61.8°C, extension for 20 s at 68°C. The resulting standard curve for *Methanosaeta* had an efficiency of 98% (R^2^=0.9826) with a method detection limit of 1x10^4^ 16S rRNA gene copies per PCR reaction. For qPCR of *Methanosarcina*, thermal cycling conditions were: initial denaturation at 95°C for 10 min, 50 cycles with denaturation for 10 s at 95°C, annealing for 20 s at 64°C, extension for 20 s at 68°C. The resulting standard curve for *Methanosarcina* had an efficiency of 94% (R^2^=0.9982) with a method detection limit of 1x10^1^ 16SrRNA gene copies per PCR reaction. qPCR cycling conditions for bacteria were: initial denaturation at 95°C for 10 min, 50 cycles with denaturation for 15 s at 95°C, annealing for 30 s at 55.4°C, extension for 20 s at 68°C. Melting curves generated at the end of the qPCR reactions were routinely examined to verify the correct amplification The resulting standard curve for Bacteria had an efficiency of 84% (R^2^=0.9826) with a method detection limit of 3x10^3^.

**Table 2:** Primers for *Methanosaeta*, *Methanosarcina*, Bacteria, and Archaea.

Target Organisms	Primer	Sequence	Concentration	Reference
*Primers used for qPCR*				
*Methanosaeta*	MS1b	5'-CCGGCCGGATAAGTCTCTTGA-3'	0.5 μM	[27]
	SAE835R	5'-GACAACGGTCGCACCGTGGCC-3'	0.5 μM	[27]
*Methanosarcina*	MB1b	5'-CGGTTTGGTCAGTCCTCCGG-3'	0.3 μM	[27]
	SAR835R	5'-AGACACGGTCGCGCCATGCCT-3'	0.3 μM	[27]
Bacteria	1114F	5'-CGGCAACGAGCGCAACCC-3'	0.5 μM	[26]
	1275R	5'-CCATTGTAGCACGTGTGTAGCC-3'	0.5 μM	[26]
*Primers used for cloning reactions*				
Bacteria	8F	5'-AGAGTTTGATCCTGGCTCAG-3'		[28,29]
Archaea	21F	5'-TACCTTGTTACGACTT-3'		[28,29]
Universal	1492R	5'-TTCCGGTTGATCCYGCCGGA-3'		[28,29]

### Clone Libraries and Phylogenetic Analysis

16S rRNA genes were amplified with forward primers specific for *Bacteria*, and *Archaea* combined with a Universal reverse primer [28,29] (Table 2). Clone libraries were constructed by using TOPO TA Cloning Kit (Invitrogen Molecular Probes, Grand Island, NY). 16S rRNA genes in clones were sequenced at the High Throughput Genomics Center at University of Washington from one end (*Bacteria*: 8F, *Archaea*: 21F or 1492R). Sequences were trimmed and assembled using Sequencher^®^ (version 4.9, Gene Codes Corporation, Ann Arbor, MI), and sequences having similarity >99% were defined as an OTU (Operational Taxonomic Unit). Taxonomic affiliation of OTU was determined by RDP Classifier [30]. All sequences were deposited in the GenBank database under accession numbers KC961776 - KC961942.

## Results

### Morphology of VLPs

**Figure 1:**
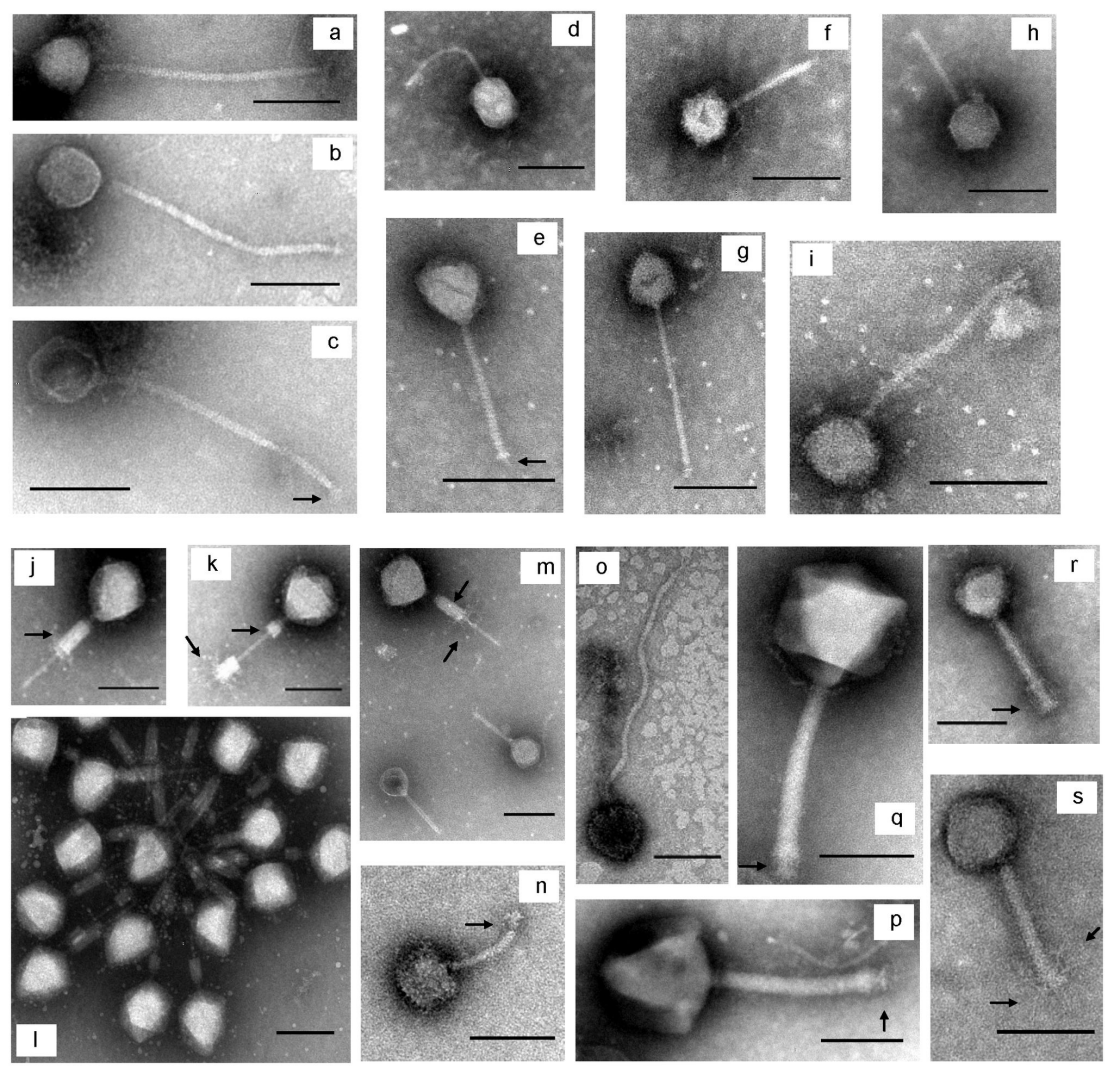
Selected transmission electron microscope (TEM) micrographs of virus-like particles (VLPs) in the reactors. VLPs in the daily-fed reactor (a-i) and the hourly-fed reactor (j-s). VLPs morphologically similar to *Siphoviridae* (a, b, c, e, g, i and o) and to *Myoviridae* (j, p, q, r and s) were observed. Scale bar represents 100 nm.

TEM images of VLPs from the reactors are shown in Figure 1. All of the observed VLPs were head-tailed morphologies, consistent with the descriptions of the order *Caudovirales* [31,32]. Within this morphology, two distinct variations were observed: isometric capsids with long noncontractile tails (characteristic of the family *Siphoviridae*; ex: Figure 1 a, b, c, e, g, i and o) in both reactors, and isometric capsids with long contractile tails (characteristic of the family *Myoviridae*; ex: Figure 1 j, p, q, r and s) only in the hourly reactor. One example of an additional morphology was observed (Figure 1d) that had a moderately elongated capsid. The only described family within the order *Caudovirales* not observed in this study was *Podoviridae*, which is characterized by short tails. Various facultative structures of VLPs were evident in the TEM micrographs (for a review of the description of facultative structures see 32). For example, tail sheaths (Figure 1 j, k and m), base plates (Figure 1 e, n and p), tail spikes (Figure 1e) and tail fibers (Figure 1 k, m, r, and s) were observed as indicated by arrows in the figures.

### Size Distribution of VLPs

**Figure 2:**
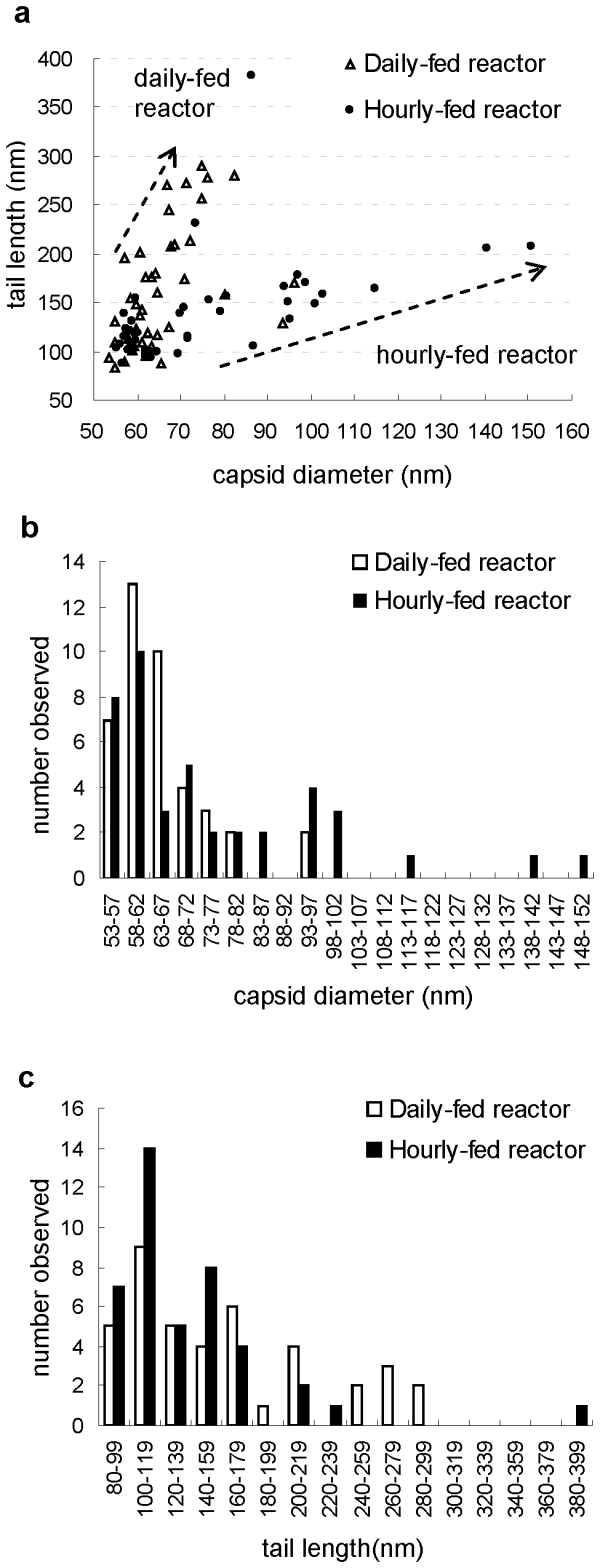
Distribution of measured capsids diameters and tail lengths. 41 virus-like particles (VLPs) from the daily-fed reactor and 42 VLPs from the hourly-fed reactor were measured. **a** XY scatter chart, **b** size of capsids: X-axis shows capsid diameters and Y-axis shows number observed, **c** tail lengths: X-axis shows tail lengths and Y-axis shows number observed.

The sizes of capsids and tails of 41 VLPs from the daily-fed reactor and 42 VLPs from the hourly-fed reactor were measured. Size distribution of VLPs varied between two reactors (Figure 2a) with smaller capsids and longer tails observed in the daily-fed reactor, and larger capsids and shorter tails in the hourly-fed reactor (Table 3).

**Table 3:** Dimensions of VLPs observed in the reactors in comparison to previously documented phage families.

reactor/phage families	Head diameter (nm)			Tail length (nm)		
	average	range	STD**^*b*^**	average	range	STD**^*b*^**
daily-fed reactor	66	54 - 96	9.6	162	84 - 289	61.4
hourly-fed reactor	76	55 - 151	22.6	137	87 - 382	51.4
*Siphoviridae**^a^***	55	40 - 97	--	191	79 - 593	--
*Myoviridae**^a^***	85	53 - 160	--	167	80 - 485	--

^***a***^ 251 total phages examined (Ackermann 1998) [32]

^***b***^ STD, standard deviation

In the daily-fed reactor, the size distribution of isometric capsid diameters showed a distribution peak in the 58 nm - 62 nm group (Figure 2b) while no clear peak was found in the distribution of tail lengths (Figure 2c). Long flexible noncontractile tails were observed. Four of these ranged between 200 nm and 220 nm and 7 tails were longer than 240 nm.

In the hourly-fed reactor, the size distribution of head diameters also showed a peak in the 58 nm - 62 nm group (Figure 2b). VLPs with extremely large capsid were also observed (e.g. Figure 1p: 140 nm and 1q: 150 nm). Most tail lengths ranged from 80 nm to 240 nm with one exception of a VLP with a 380 nm tail (Figure 2c and Figure 1o). The distribution of tail lengths formed two peaks, one with tail length ranging from 100 nm to 119 nm and the other with tail length between 140 nm and 159 nm.

### Concentration of VLPs using EFM

**Figure 3:**
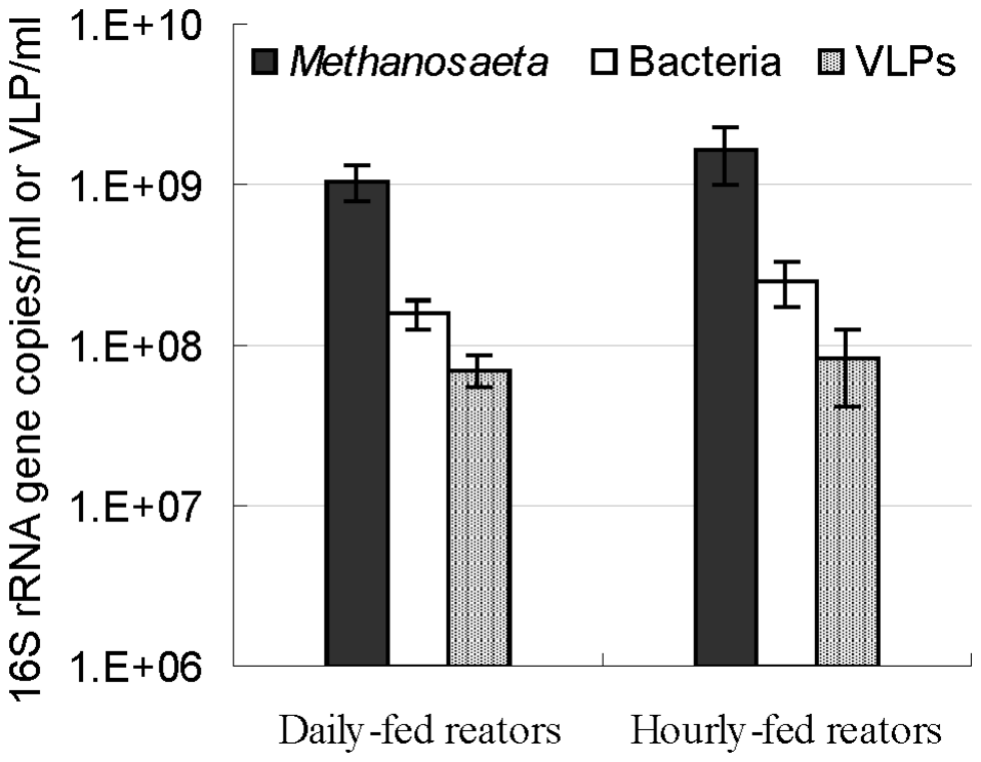
Concentrations of *Methanosaeta*, Bacteria and virus-like particle (VLP) in the daily-fed and hourly-fed reactors. Values are the average of 6 samples for *Methanosaeta* and Bacteria, and 10 (daily-fed) and 7 (hourly-fed) samples for the VLP. Standard deviations were indicated by error bar.

The average concentrations of VLPs in the reactors quantified using EFM are shown in Figure 3. Variation of VLP concentrations was low over the course of a week and before and after automated feeding. The concentration of VLPs were similar in the hourly-fed reactor (8.4 ± 4.3×10^7^ VLPs/ml) than in the daily-fed reactor (7.1 ± 1.5×10^7^ VLPs/ml). Variation shows the deviation among multiple samples collected from the reactors at different times, and no significant difference indicated between the two (T-test, P = 0.05). 

### Genome size of VLPs

**Figure 4:**
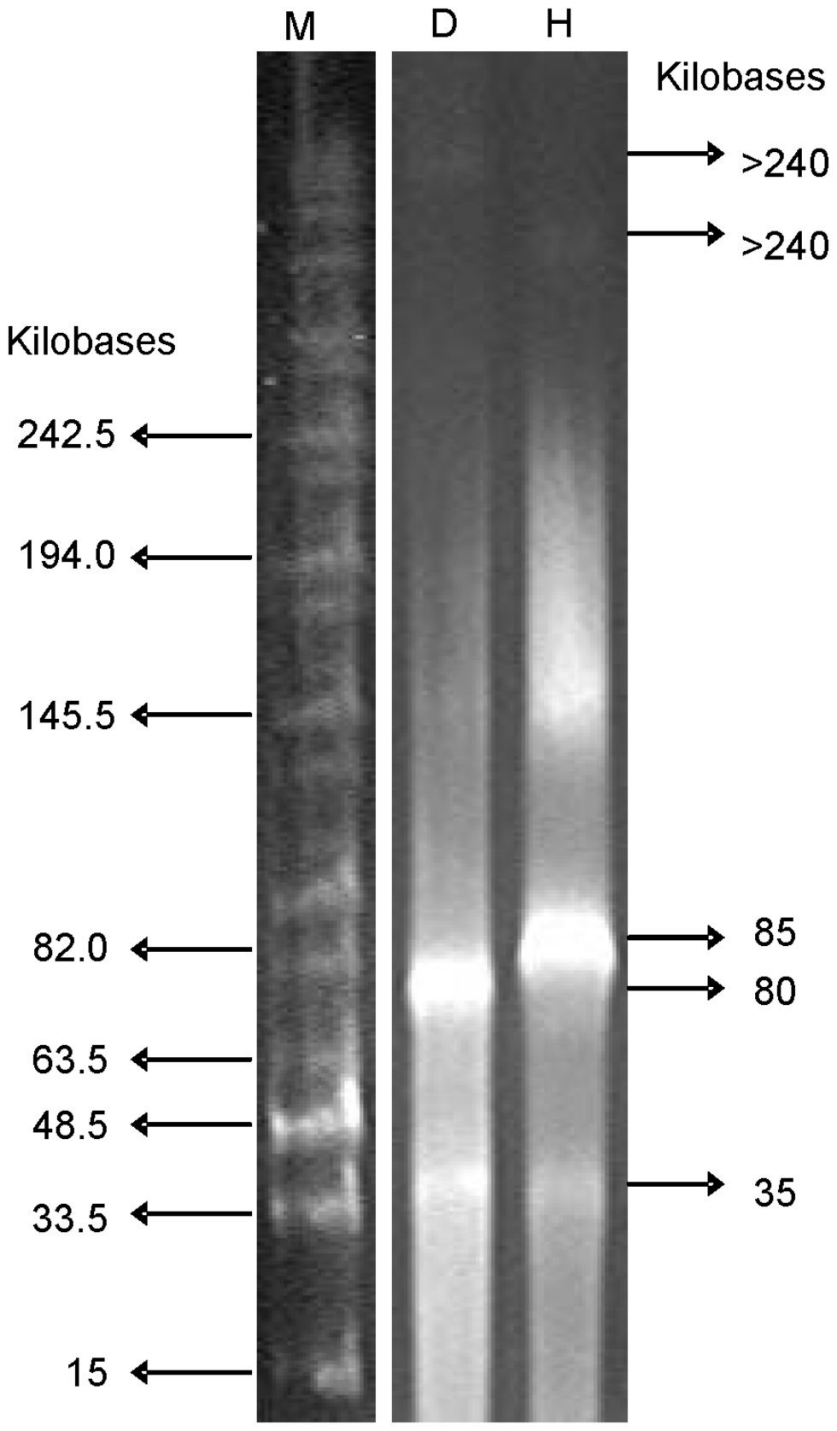
Pulsed field gel electrophoresis (PFGE) of viral genomes from reactors.

Viral genome size distributions for the daily-fed and hourly-fed reactors are shown in the PFGE results (Figure 4). Different banding patterns were observed between the two reactors. Three bands (two major and one minor) were discovered in each reactor. The major (brightest) bands in the daily-fed and hourly-fed reactors were observed at approximately 80 and 85 kbp, respectively. The second brightest band was observed at approximately 35 kbp for both reactors. A third dim band in each reactor was observed at a higher molecular weight range (>240 kbp). Additional background smearing observed in the hourly-fed reactor, especially around 145 kbp may suggest DNA from multiple virus species with similar genome sizes.

### Performance of the Acetate-fed Reactors

Both the daily-fed and hourly-fed reactors had neutral pH and consistent methane production prior to sampling. Monitoring of acetate concentrations, gas production and gas composition in the reactors showed that nearly all of the acetate was converted to methane (calculations data not shown). Neither reactor had any upsets or failure during the course of the study.

### Dominant Reactor Microorganisms

**Figure 5:**
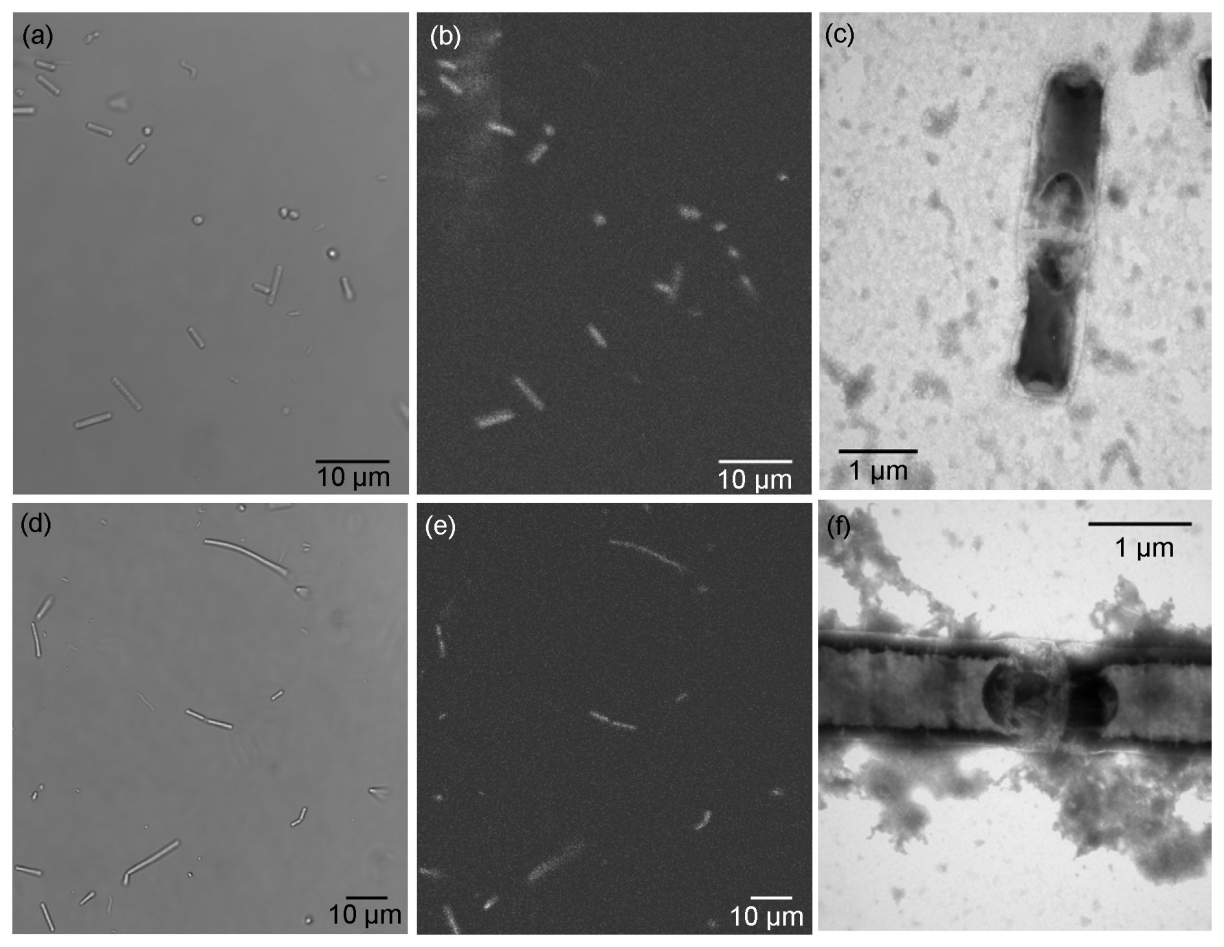
Micrographs of enriched cultures in the daily-fed and hourly-fed reactors. Micrographs (a-c) are from daily reactor and (d-f) are from hourly reactor. (a) and (d) are transmitted light bright field images. (b) and (e) are fluorescence micrographs. (c) and (f) are transmission electron microscope micrographs.

Three independent methods were used to demonstrate the dominance of *Methanosaeta* in the reactors including microscopy, qPCR and sequence recovery. Filamentous cells composed of several single flat-end rods (0.8-0.9 × 1.8-3.3 μm) were dominant in both reactors (Figure 5 a, c, d and f). These rods were non-motile and generally grew as long threads (3-50 μm). Autofluorescence characteristic of methanogenic Archaea was confirmed in the dominant microbial populations in the samples (Figure 5 b and e). The rod-shapes observed are consistent with *Methanosaeta* as described elsewhere [20,33,34]. Coccoidal morphotypes were observed with much lower frequency, some of which were autofluorescent.

*Methanosaeta* qPCR signals dominated the reactors (Figure 3), while *Methanosarcina* was not detected (detection limit in the extract sample of 3x10^2^ copies/ml of reactor effluent). *Methanosaeta* 16S rRNA gene copy concentrations were ~7× greater than total bacterial gene copy concentrations. *Methanosaeta* concentrations were not statistically different in the two reactors, while bacterial concentrations were higher in the hourly-fed reactor (T-test, P = 0.05).

Phylogenetic affiliation of archaeal and bacterial 16S rRNA genes from the two reactors as determined by clone sequencing are summarized in Table 4. In corroboration of the qPCR and microscopic evidence, the most frequently detected archaeal phylogenetic group was *Methanosaeta* in both reactors. One clone (out of 73 archaeal clones) in the daily-fed reactor aligned with the genus *Methanosphaera* (a Coccoidal-shaped microbe). No clone sequences from the group *Methanosarcina* were detected.

**Table 4:** Phylogenetic analysis of 16S rRNA gene in the daily-fed and hourly-fed reactors.

Clone Library	Taxon**^*a*^**	No. of OTU**^*b*^**	No. of clone	Daily-fed (%)	Hourly-fed (%)
Archaea-21F	*Methanosaeta*	4	36	96.4	100.0
	*Methanosphaera*	1	1	3.6	-
Archaea-1492R	*Methanosaeta*	4	36	100.0	100.0
Bacteria	*Proteobacteria*	6	43	47.8	44.7
	*Firmicutes*	7	21	19.6	25.5
	*Bacteroidetes*	5	13	13.0	14.9
	WWE1	2	7	2.2	12.8
	minor groups**^*c*^**	6	9	17.4	2.1

^***a***^ Taxon was determined by RDP classifier [30]; Archaea was grouped at the genus level and Bacteria was group at phylum level

^***b***^ OTU, Operational Taxonomic Unit

^***c***^ Clones with either 2 or less representatives in the library were from the groups *Spirochaetes*, *Chloroflexi*, *Lentisphaerae*, SR1 and unclassified bacteria.

For the bacterial domain, the most frequently observed clones in both reactors were from the phyla *Proteobacteria*, *Firmicutes*, and *Bacteroidetes* (Table 4). While other groups were consistent between the two reactors, more clones for the WWE1 (for a description of phylum WWE1 see 35) were obtained from the hourly-fed reactor. Of the *Proteobacteria*, the *Arcobacter* genus was represented by 35 clones, consisting of 45.7% of the *Proteobacteria* in the daily-fed and 29.8% in the hourly-fed reactors (data not shown).

## Discussion

### VLPs in Enriched Reactors

VLPs have been previously reported in anaerobic digesters [15,16]. However to the best of our knowledge, this is the first time that VLPs associated specifically with acetate-fed methanogenic consortia enriched in *Methanosaeta* have been characterized. Anaerobic digester sludge from municipal wastewater treatment plants have had higher reported VLP concentrations (e.g. 2.4×10^10^ VLPs/ml [16]) likely associated with higher microbial diversity in these systems compared to the study enrichment reactors. Still, the prevalence of *Siphoviridae* observed in the study reactors was consistent with Ackermann’s 2007 survey that showed this virus familyto be the most common tailed-phage documented in published electron micrograph images irrespective of the habitat. *Siphovirdae* were also the most abundant identified morphotype (16%) in a previously studied methanogenic digester treating wastewater from a beer brewery [15]. Observation of this common viral family is particularly interesting because although some archeoviruses have been reported to exhibit exceptionally complex morphotypes (e.g. linear, fusiforms, droplet and bottle shapes) [12,36], currently characterized viruses of methanogens typically have head-tail morphologies [37–39].

Several observations demonstrate that virus populations between two reactors differed. First, VLPs morphologically similar to *Myoviridae* were only found in the hourly-fed reactor (Figure 1). Second, different dominant VLP capsid sizes and tail lengths were observed in the two reactors (Figure 2a), a parameter thought to be highly conserved and uniform for individual virus species [32]. Third, PFGE banding patterns suggested that the viruses in the two reactors had different genome sizes (Figure 4, e.g. virus with 80 kbp genome in the daily-fed reactor and virus with 85 kbp in the hourly-fed reactor). Different virus populations may have developed in the reactors due to several contributing factors such as feeding schedule, HRT, re-inoculation of the daily-fed reactor, natural population drift in each reactor caused by unique “virus-host arm races”.

### Enriched Cultures and Microbial Communities

One genus of the acetoclastic methanogens, *Methanosaeta*, was dominant in both reactors. Higher periodic acetate concentrations and shorter HRT in the daily-fed reactor was designed to favor *Methanosarcina* due to its higher growth rate (k) and lower acetate affinity (higher *K*s) than *Methanosaeta* and *Methanosarcina* was historically dominant in the daily-fed reactor as previously reported [22]. However, dominance shifted from toward *Methanosaeta* by year 2007. Re-inoculation of the daily-fed reactor with sludge from municipal anaerobic digesters failed to reestablish *Methanosarcina*, as evidenced by qPCR, microscopic, and sequencing results presented in this work. The loss of *Methanosarcina* in the daily-fed reactor remains unexplained, but allowed comparison of VLP communities in two reactor systems that shared a dominant microbial population.

While the Bacteria were less prevalent than the *Methanosaeta*, their phylogeny still reveal interesting insights into the reactor microbial communities. The phyla *Proteobacteria*, *Firmicutes* and *Bacteroidetes* detected in the reactors have previously been reported as core microbial groups in several full-scale mesophilic anaerobic digesters [40] and in mesophilic bovine serum albumin-fed reactors [41]. One of the OTU detected in this study (containing 35 clones) within phylum *Proteobacteria* was assigned to genus *Arcobacter* and was most frequently detected in both the daily-fed (45.7%) and hourly-fed (29.8%) reactors (data not shown). The closest related sequence (NCBI accession number: GQ136513.1, 100% sequence coverage and similarity) to this OTU was a clone also found in an acetate enriched digester sample, implying that an anaerobic acetate enriched niche may be a favorable habit for *Arcobacter*. Sequences assigned to WWE1 were observed more frequently in the hourly-fed reactor, while the sequences affiliated with minor groups were more dominant in the daily-fed reactor, indicating different bacterial communities between the two reactors. Most of the bacterial 16S rRNA gene sequences recovered did not closely affiliate to any known isolated culture (similarity <97%) but were similar to the clones found in the anaerobic digesters [40–44], methanogenic consortium and landfill leachate [45] suggesting these anoxic systems may contain many bacterial species with yet unconfirmed metabolic roles.

### VLPs and Enriched Cultures

Observations of persistent VLP populations in the reactors suggest that they are actively propagating in the system. Because viruses are obligate parasites, they only reproduce when their corresponding hosts (Bacteria or methanogenic Archaea in this study) are present. The feed for the reactors was prepared aseptically and evaluated by EFM to confirm that VLPs were not present. VLPs that entered the reactors with the anaerobic digestion sludge used to inoculate the reactors is not mathematically predicted to have persisted in the reactors due to the extended time since the last re-inoculation (>3 years prior to the study) in comparison to the systems’ retention times. Thus, replication of the observed VLPs was the most likely explanation for observation of VLP persistence in the system.

While replication of the virus population was supported by the data, the virus-to-bacteria ratio (VBR) values were lower in the study reactors than is often reported. The ratio of VLP concentrations to the prokaryotic cells (*Methanosaeta* and bacteria) concentration was 0.123 in the daily-fed reactor and 0.093 in the hourly-fed reactor (assuming 2 and 3.8 16S rRNA genes copies per organism for *Methanosaeta* [46] and Bacteria [47], respectively. In contrast to the study calculation, when VBR has been used to study relationships between viruses and bacteria in many natural aquatic ecosystems, the number of viruses are higher than the number of bacteria with typical VBRs ranging from 3 to 10 in aquatic ecosystems [48]. Assumptions used to estimate microorganism concentrations from 16S rRNA copy number may partially contribute to lower VBR values, particularly because the numbers of 16SrRNA genes in a chromosome can vary among species of the same genus and some methanogens have recently been reported to contain more than one chromosome [49] (though not yet studied for slow-growing *Methanosaeta*). However, lower VBR values similar to this study’s results have been reported in an oligotrophic lake (0.03-0.7) [50] and in rhizosphere soil (0.04) [51]. VBR values in Archaea-dominant environments have not been reported. 

Finding of low VLPs concentration in the two *Methanosaeta*-dominant reactors studied here may due to several reasons. First, viruses in reactors might prefer lysogenic or pseudolysogenic [52] life cycles, in which viruses persist inside host cells and thus were not counted using the study methods. Second, it has been shown that the VBR is higher in nutrient-rich, more productive environments [48]. However, the acetate-fed methanogenic reactors in this study are not classified as productive environments for viruses. Under anaerobic conditions, limited energy is gained by slow growing *Methanosaeta* when acetate is converted to methane, and only limited substrate (e.g. vitamins) was available for the growth of bacteria (although metabolic roles of bacteria in reactors is unknown). Third, a positive correlation between VBR and host community diversity has been previously observed [53], suggesting that the VBR may be an indicator of host community diversity. For the reactors in the current study, *Methanosaeta* was found to be dominant in the daily-fed and hourly-fed reactors and low diversity of microbial communities was observed, which may explain with the low VBR observed in two enriched reactors.

Despite persistent VLP presence in both *Methanosaeta* dominant reactors, upset or failure of these methanogenic reactors was not observed over the study period. This might be explained by the presence of temperate viruses, chronic infections or co-evolution of virus and host populations in reactors. Limited energy conditions and a slow growing host under anaerobic conditions may be favorable for lysogenic or pseudolysogenic life cycles. Additionally, some crenarchaeal viruses have been found to be chronically produced without host cells lysis [12,54,55]. Although viruses of acetoclastic methanogens have not yet been reported, this hypothesis seems less likely because currently identified head-tail morphotypes, such as those observed in this study, have previously been reported as lytic [37,39]. Finally, an evolutionary arms race between viruses and hosts could result in minor changes in population structure without the changing of community stability [56]. Nevertheless, this theory requires more investigation for methanogenic habitats.

Viral-host dynamics for the archaea are complex. Further understanding of the viral life strategies (e.g. lysis, lysogeny, and pseudolysogeny) and coevolution with host will lead to improved understanding of the impact of acetoclastic archeoviruses on microbial ecology of anaerobic digesters.
